# Role of the Surgical Approach in the Treatment of Eagle Syndrome

**DOI:** 10.1055/s-0043-1776717

**Published:** 2024-03-15

**Authors:** Sahil Kapoor, Ayushi Gupta, Sneha Satya, Poonam K Saidha, Urvi Saini, Ankesh Singh

**Affiliations:** 1 Department of Otorhinolaryngology, Faculty of Medical & Health Sciences, SGT University, Gurugram, Haryana, India; 2 Department of Otorhinolaryngology, ESIC Hospital & PGIMSR Basaidarapur, New Delhi, India; 3 Department of Anesthesia, Faculty of Medical & Health Sciences, SGT University, Gurugram, Haryana, India; 4 Department of Otorhinolaryngology, Rama Medical College, Kanpur, India; 5 Department of Otorhinolaryngology, ESIC Medical College & Hospital, Faridabad, Haryana, India; 6 Department of Psychiatry, All India Institute of Medical Sciences (AIIMS), New Delhi, India

**Keywords:** Eagle syndrome, elongated styloid process, otalgia, styloidectomy

## Abstract

**Introduction** Eagle syndrome is a rare and an often misdiagnosed entity in otorhinolaryngology.

**Objective** To determine the efficacy of the surgical treatment for Eagle syndrome.

**Methods** The present prospective study included 25 patients who presented with complaints of pain in the throat, ear, and neck, as well as difficulty and/or pain while swallowing; they were assessed for Eagle syndrome. As per patient profile, we performed a clinical assessments along with orthopantomograms (OPGs), three-dimensional computed tomography (3D CT) scans, and cone beam computed tomography (CBCT). Pain was assessed pre- and postoperatively through the Numerical Rating Scale-11 (NRS-11), whose score ranges from 0 to 10. Microscopic tonsillo-styloidectomy was performed in cases in which the conservative treatment failed to relieve pain.

**Results** The mean age of the entire study population was of 36.08 ± 7.19 years, and the male-to-female ratio was of 1.08:1. Referred otalgia was the commonest (44%) complaint. Radiologically, out of 25 patients, 20 patients presented elongated styloid processes. The longest symptomatic styloid process measured radiographically was of 64.7 mm while the shortest was of 28.2 mm. Out of 20 patients, 12 underwent surgery. The postoperative pain assessment through the NRS-11 was performed on day 0 (3.83 ± 0.83), day 7 (1.5 ± 0.52), week 4 (0.5 ± 0.52), and week 12 (0.41 ± 0.51). By 12 weeks, 7 patients were symptom-free, while 5 patients still reported mild pain.

**Conclusion** Eagle syndrome associated with an elongated styloid process is not a rarity, but it often goes undiagnosed. Microscopic tonsillo-styloidectomy shows excellent results in the management of patients with Eagle syndrome.

## Introduction

Eagle syndrome (ES) is a rare and an often-misdiagnosed entity in otorhinolaryngology. Watt Weems Eagle 1 first described the condition, which is also called stylalgia, as a pain syndrome due to elongation and/or ossification of the styloid process and/or impingement of the stylohyoid ligament on the surrounding neurovascular structures. Its clinical presentation varies from throat pain, ear pain or referred otalgia, sensation of a foreign body in the throat, neck pain or headache, and dysphagia. 2 The exact etiopathogenesis of ES still remains unclear. However, several theories have been suggested, such as congenital elongation due to the persistence of an embryonic cartilaginous outgrowth, calcification of the stylohyoid ligament, and formation of bone tissue at the insertion of the ligament. Other causes, such as genetic predisposition, trauma, early menopause, local stretching, and scarring after tonsillectomy leading to insertion tendinosis of the stylohyoid ligament have also been postulated. 3

The normal length of the styloid process is considered to be of approximately 20 mm to 30 mm; in adults, lengths greater 30 mm are considered abnormal. 4 The incidence of elongated styloid process ranges from 6% to 7% of the general population, but only 4% to 10% are symptomatic, with a male-to-female ratio of 1:3. 4 5 A study 6 has reported that radiographic evidence of an elongated styloid process is found in 19.4–52.1% of the Indian population. Variations in length, angulation, and other morphological features of the styloid process among individuals can lead to the development of a constellation of symptoms. 7

Several imaging modalities can be used for the diagnosis, such as X-ray on lateral skull view, Towne panoramic view etc. However, the orthopantomogram (OPG) and three-dimensional computed tomography (3D CT) contribute to the accurate diagnosis of ES. 5 8 Cone beam computed tomography (CBCT) is a new and advanced imaging technique which generates high-resolution 3D images with a lower absorbed dose and is cost-effective compared with the CT scan. 8

The treatment strategies depend on the severity of the symptoms. The first-line management is conservative, and it includes non-steroidal anti-inflammatory drugs (NSAIDs), analgesics, anticonvulsants, antidepressants, and use of local infiltration of anesthetics/corticosteroids in the tonsillar fossa. 5 However, in case of failure of the conservative treatment to provide long-term satisfactory benefits, surgery has been advocated as the definitive treatment for ES. Styloidectomy by the intraoral or external transcervical approaches aims at shortening the length of the styloid process to relieve the pain. 5 9 However, some degree of pain may persist in certain patients even after surgical removal. To date, the literature 10 considers surgery the treatment of choice, with the highest success rates. The length of the styloid process is measured from the base of the skull to the hyoid bone. In most cases, it is not the elongated styloid process, but the calcified stylohyoid ligament, which, during surgery, tends to retract once the surrounding fibers are released, thus leaving the length of the styloid process shorter than what appears on the radiological image. Further shortening and removal of the elongated styloid process can be performed superiorly until the base of the skull. Hence, the aim of the surgery is to remove and/or shorten as much as possible the length of elongated styloid process.

The present study was undertaken to assess the pain score and determine the efficacy of the surgical treatment of patients diagnosed with ES.

## Objective

1. To evaluate the efficacy of the surgical treatment by an assessment of pain in patients with ES using the Numerical Rating Scale-11 (NRS-11) pain scoring system.
2. To assess the prevalence of referred otalgia as a presenting complaint in patients with stylalgia.

## Methods

**Fig. 1:**
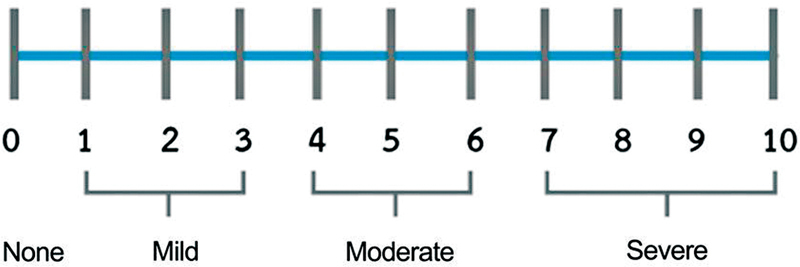
NRS-11 pain assessment scale.

A prospective study was performed over a period of 8 months in the Department of Ear, Nose, and Throat (ENT) of a tertiary care hospital in Haryana, India, among patients complaining of pain in the throat, ear, and neck, as well as difficulty and/or pain while swallowing. The study included 25 patients, 20 of whom with a diagnosis of ES. Complete history was taken along with the performance of an ENT examination, which included digital palpation of the styloid process in the tonsillar fossa on the affected side, which induced pain. Local infiltration of 2% lignocaine in the anterior tonsillar pillar of the affected side relieved the pain, thus confirming the clinical diagnosis. Further correlation and confirmation of ES were performed through an OPG and 3D CT scan; a few patients were also submitted to CBCT. Pain was assessed through the NRS-11 ( Fig. 1 ), with the following scores: 0–no pain; 1-3, mild pain; 4-6–moderate pain; and 7-10–severe pain.

All patients were submitted to two months of conservative treatment, with a combination of antidepressant and anticonvulsant drugs along with anti-inflammatory drugs. Microscope-assisted tonsillo-styloidectomy via the intraoral approach was performed under general anesthesia: the length of elongated styloid process was reduced to a minimum and the remaining was removed, thus alleviating the pressure impingement on the surrounding neurovascular structures.

### Surgical Procedure

**Fig. 2:**
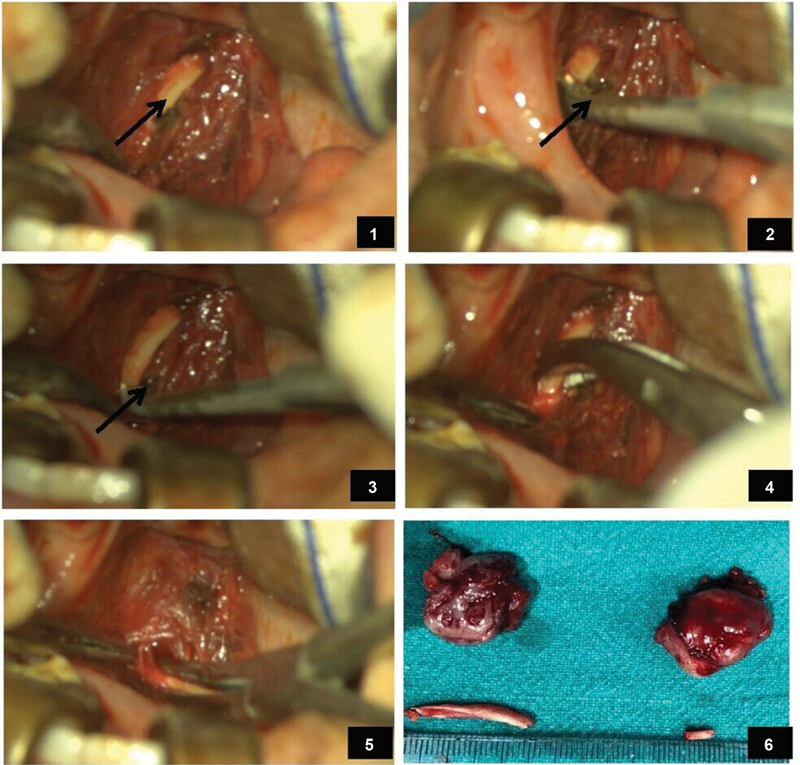
Microscope-assisted tonsillo-styloidectomy: ( **1** ) visualization of the elongated styloid process (arrow) after removal of the tonsil and separation of superior constrictor fibers; ( **2,3** ) styloid curette (arrow) hooked on the tip of the styloid process, soft tissue attachments stripped off and broken; ( **4,5** ) broken styloid process mobilized by long curved artery forceps and removed; ( **6** ) bilateral tonsillo-styloidectomy specimens after removal.

The surgery was performed under general anesthesia. In the Rose position, microscope-assisted tonsillectomy was performed by dissection and the snare method. After removal of the tonsil, the sharp tip of the styloid process was palpated in the tonsillar fossa, and the overlying superior constrictor fibers were separated with use of bipolar cautery and long artery forceps to expose the styloid process. Using a specially designed styloid curette, the soft tissue attachment of the styloid was stripped off, and the process was broken as close to the base of skull as possible and, with help of a long curved artery forceps, the broken part of the styloid was grasped, mobilized, and removed, hence reducing its length ( Fig. 2 ). After achieving hemostasis, 2–0 VICRYL suture (Ethicon, Inc., Raritan, NJ, United States) was used to approximate the separated fibers of the superior constrictor muscle to avoid creating any dead space and the possible complication of deep neck space infection after surgery. In bilateral cases, a similar procedure was repeated on the opposite side.

All patients were followed up for three months postoperatively. Pain assessment (through the NRS-11) was performed after 1, 4, and 12 weeks.

### Statistical Analysis

The statistical analysis was performed with the IBM SPSS Statistics for Windows (IBM Corp., Armonk, NY, United States) software, and the results were calculated using the one-way analysis of variance (ANOVA) test, the paired t-test, and coefficient correlations. Values of *p* < 0.05 were considered statistically significant.

## Results

**Fig. 3:**
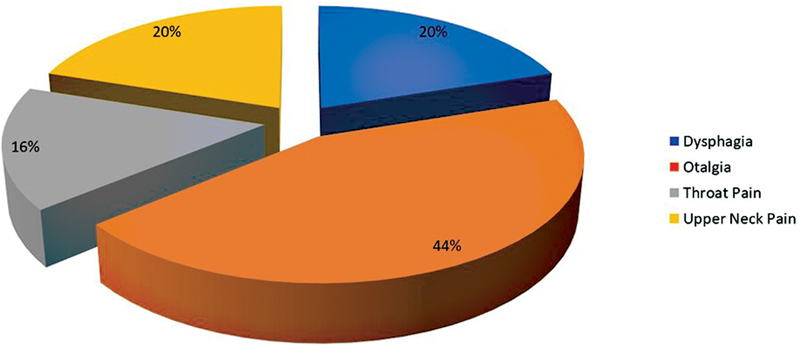
Distribution of presenting complaints.

**Fig. 4:**
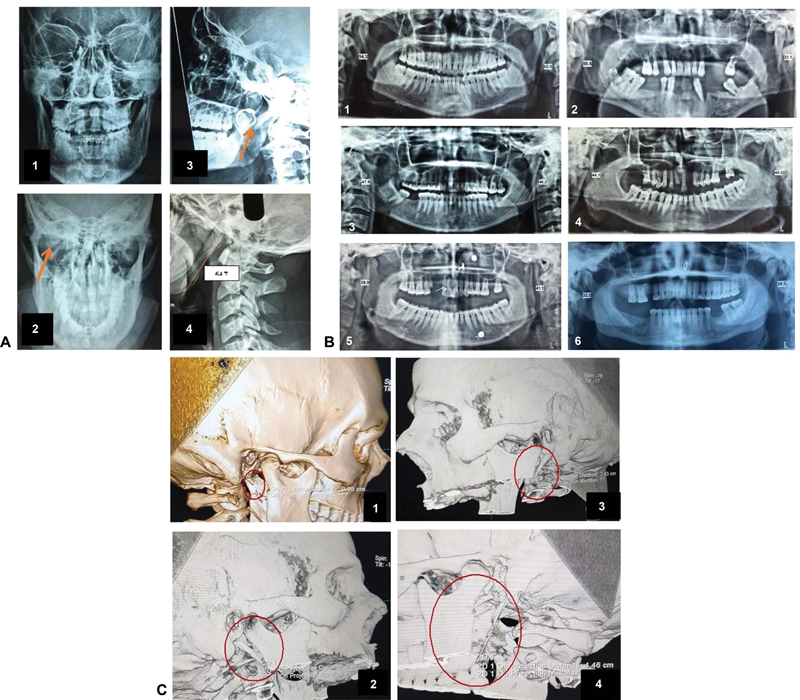
( **A** ) X-ray of the skull: Towne view ( **1,2** ) and lateral view ( **3,4** ). ( **B** ) Orthopantomogram (OPG) showing the measurements of the right (R) and left (L) elongated styloid processes: ( **1** ) R = 54.3 mm; L = 60.9 mm; ( **2** ) R = 50.5 mm; L = 31.1 mm; ( **3** ) R = 47.6 mm; L = 46.2 mm; ( **4** ) R = 44.1 mm; L = 48.5 mm; ( **5** ) R = 39.9 mm; L = 41.1 mm; ( **6** ) R = 32.2 mm; L = 28.5 mm. ( **C** ) Computed tomography scan with three-dimensional reconstruction of the skull: ( **1,2** ) lengths of normal styloid processes: 0.95 cm and 2.43 cm respectively; ( **3,4** ) elongated styloid processes measuring 2.66 cm and 4.46 cm respectively.

The study sample was composed of 25 subjects: 13 male patients (52%) and 12 female patients (48%) with a mean age of 36.08 ± 7.19 years. The commonest presenting symptoms were referred otalgia (44%), upper neck pain (20%), dysphagia (20%) and throat pain (16%). Most of the patients complained of more than one symptom ( Fig. 3 ). Based on the clinical examination and the evidence obtained from the radiographs on the lateral skull and Towne views ( Fig. 4A ), the OPG ( Fig. 4B ), the 3D CT scans or the CBCT ( Fig. 4C ), 20 out of 25 patients presented elongated styloid processes and were diagnosed with ES. The remaining 5 patients were diagnosed with chronic tonsillopharyngitis ( *n* = 2), chronic laryngopharyngeal reflux ( *n* = 1), and temporomandibular arthralgia ( *n* = 2).

**Fig. 5:**
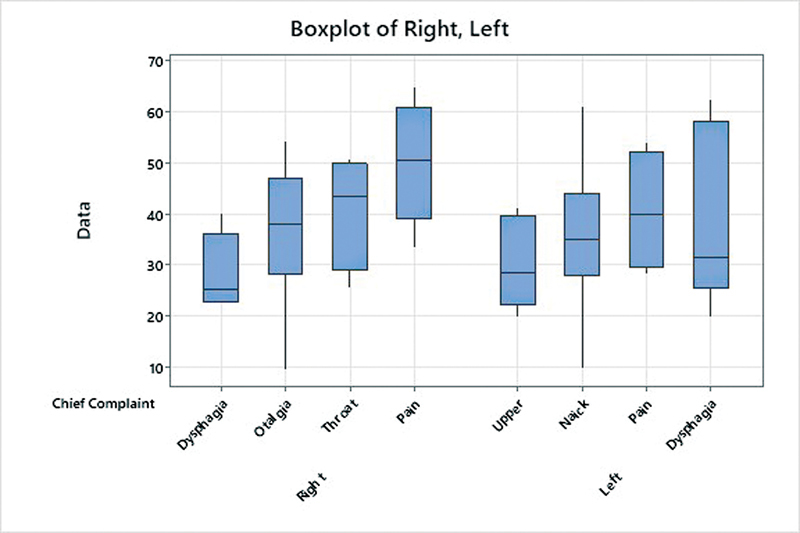
Comparison of the length of the right and left styloid processes with chief complaints, as shown in a box and whisker plot.

Among the 20 ES patients, there were 35 symptomatic styloid processes; stylalgia was bilateral in 15 patients (75%) and unilateral in 5 patients (25%). Radiologically, the longest symptomatic styloid process measured 64.7 mm while the shortest, 28.2 mm. Fig. 5 illustrates the comparison of the length of the styloid process (on the right and left sides) with the chief complaints of the patients, which was not statistically significant.

Pain was assessed on the first visit to the ENT Outpatient Department through the NRS-11, and 17 patients had severe pain, 6 had moderate pain, and 2 had mild pain. The correlation of severity of the pain with the mean length of the styloid process (on the right and left sides) was found to be statistically significant, as shown in Table 1 . All the patients ( *n* = 25) were managed conservatively for two months for symptomatic pain relief.

**Table 1:** Degree of pain in right- and left-sided styloid elongation

Pain score category	Right (mean ± standard deviation)	Left (mean ± standard deviation)
**Mild (** ***n*** ** = 2)**	16.0 ± 9.2	17.3 ± 10.3
**Moderate (** ***n*** ** = 6)**	30.9 ± 7.7	29.4 ± 9.9
**Severe (** ***n*** ** = 17)**	43.7 ± 10.4	40.6 ± 11.6
***p*** **-value**	0.001	0.013

Out of the 20 cases of ES, 12 patients (60%) underwent surgery ( intraoral microscope-assisted tonsillo-styloidectomy), as there was no pain relief with the conservative management. The longest styloid, which measured 64.7 mm radiologically, was reduced in length surgically, and the resected part measured 38 mm. The styloid processes were located anteriorly in 5 cases; hence, they were difficult to locate intraoperatively. The postoperative pain assessment through the NRS-11 was performed on day 0 (3.83 ± 0.83), day 7 (1.5 ± 0.52), week 4 (0.5 ± 0.52), and week 12 (0.41 ± 0.51). However, the pain score on day 0 can be considered a confounding factor (due to immediate postoperative surgical site pain). Table 2 describes the comparison and correlation between the pre- and postoperative pain scores. Out of the 12 patients, 6 (50%) were symptom-free (NRS-11 score of 0) 4 weeks postoperatively, and 6 (50%) presented mild pain (NRS-11 score of 1). By 12 weeks, 7 patients were symptom-free while 5 patients still reported mild pain.

**Table 2:** Pre- and postoperative pain assessments in cases of Eagle syndrome

Age	Sex	Laterality	Pain score	Pain Score	Postoperative pain score (NRS-11)				Pain Score
**(in years)**			**(NRS-11)**	**(Category)**	**Day 0**	**Day 7**	**4 weeks**	**12 weeks**	**(Category)**
45	M	BL	6	Moderate	4	2	0	0	None
42	M	BL	9	Severe	5	2	1	0	None
39	F	BL	7	Severe	4	1	1	1	Mild
36	F	BL	8	Severe	3	1	0	0	None
34	M	BL	9	Severe	5	2	1	1	Mild
29	F	BL	8	Severe	3	1	1	1	Mild
30	M	UL	7	Severe	3	2	1	1	Mild
48	M	BL	9	Severe	4	1	0	0	None
29	M	UL	8	Severe	5	1	1	0	None
29	F	UL	8	Severe	3	2	0	0	None
36	F	BL	9	Severe	3	2	0	0	None
32	M	BL	9	Severe	4	1	0	1	Mild
**Median (IQR: Q1, Q3)**			8 (7.7, 9)		4 (3, 4.2)	1.5 (1, 2)	0.5 (0, 1)	0 (0, 1)	−
**Mean difference**			−		4.2	6.5	7.5	7.6	−
***p*** **-value**			−		0.0005	0.0005	0.0005	0.0005	−

Abbreviations: BL, bilateral; F, female; IQR, interquartile range; M, male; NRS-11, Numeric Rating Scale-11; UL, unilateral.

Note: Wilcoxon Matched-Pairs Test is the Statistical Test Used in this Table.

The remaining 8 patients continued with the conservative management, 5 of whom reported significant symptomatic improvement with medications, while 3 refused them because of fear of anticipated surgical risks.

Long-term follow up of all the patients was difficult, as many of them lived far away from where the study was conducted.

## Discussion

Stylohyoid syndrome, or ES, is a complex entity that is often missed, especially when dealing with patients with referred otalgia. The worldwide incidence of elongated styloid process is of 4% to 7%. However, ∼19.4% to 52.1% of the population of India was found to have radiographic evidence of elongated processes, with the highest (52.1%) number of cases observed in Northern India. 11 The overall incidence remains variable, as no systematic and reliable measurement has been described yet.

The syndrome is more frequent in women than in men, and it usually occurs in older age groups. In the present study, the male-to-female ratio was of 1.08:1, and the mean age of the sample was of 36.08 ± 7.19 years. Bhuyan et al. 12 reported that the youngest patient in their study was 28 years of age, and the oldest, 55 years (mean age: 42.5 years).

The actual cause of the elongated styloid process is difficult to identify as it can either be due to partial or complete calcification (of genetic origin) of the stylohyoid ligament and growth of osseous tissue at insertion of stylohyoid ligament or it may be due to trauma or early-onset menopause, as suggested in some studies. 5 The characteristic dull and nagging pain of an elongated styloid process that worsens during deglutition and can be reproduced by palpation of the tonsillar fossa is the hallmark. This can be due to compression of the glossopharyngeal nerve fibers, irritation of the pharyngeal mucosa, and fracture of the ossified stylohyoid ligament and, in cases of posttonsillectomy, to stretching and fibrosis of the fifth, seventh, ninth, and tenth cranial nerve fibers. 5

A wide variety of symptoms have been associated with elongated styloid processes, such as otalgia, sensation of a foreign body in the throat, dysphagia, and cervicofacial pain. 5 In the present study, referred otalgia was the commonest presenting symptom, followed by upper neck pain, dysphagia, and throat pain, while, in a study by Bhuyan et al., 12 pain and sensation of a foreign body in the throat was the main presentation, followed by otalgia, headache, and dysphagia.

Elongated styloid processes were observed in 20 out of 25 patients in the present study; bilateral cases (75%) were commoner than unilateral cases, a finding that is in accordance with the study by Hajare et al. 13

The diagnosis of ES is established based on clinical assessment and radiological imaging. In the present study, OPG, 3d CT scans, and CBCT were performed for an accurate assessment of the length and angulation of the styloid process and its relationship with adjacent anatomical structures, providing a roadmap for surgical planning. 14 Out of 35 styloid processes, the longest measured 64.7 mm, while the shortest, 28.2 mm; the average length was of 43.8 mm. In the study by Hajare et al., 13 the lengths ranged from 35 mm to 50 mm.

There are various pain scales available for the assessment of pain intensity, such as the NRS, the Verbal Rating Scale (VRS), or the Visual Analogue Scale (VAS), which is the most commonly used. Although the NRS and VAS are equally efficient for pain assessment, the NRS is preferred in chronic pain conditions, because of its ease of use and standardized format. There are many variations of the NRS available, the preferred being the NRS-11. 15 16

Malik et al. 17 used the VAS in their study, while, in the present study, all patients ( *n* = 25) were assessed pre- and postoperatively through the NRS-11 and were managed conservatively for symptomatic pain relief with NSAIDs. 17 The diagnosed cases of ES ( *n* = 20) were treated conservatively with pregabalin 75mg/day for two weeks: 8 patients presented significant pain relief and continued with the treatment for another two-three weeks. The remaining 12 patients, who presented no relief after weeks of medical therapy, were submitted to surgery.

**Fig. 6:**
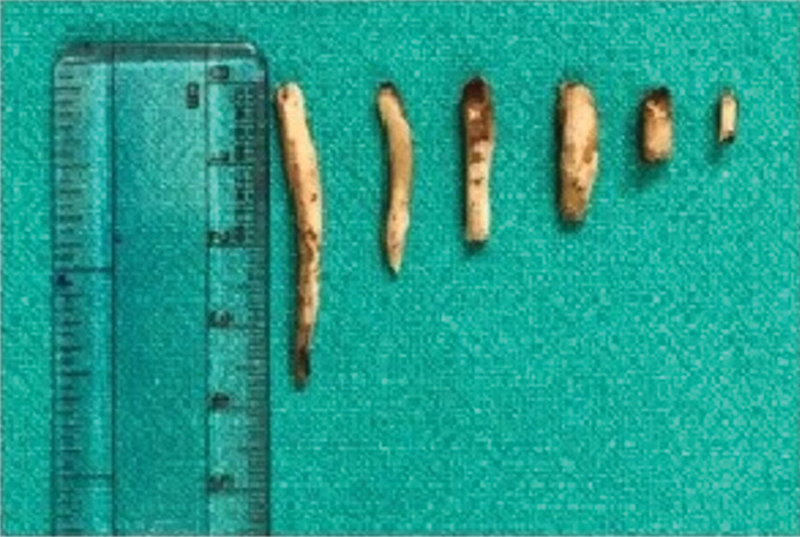
Lengths of the styloid processes removed, which ranged from 3.8 cm to 0.5 cm.

Both intraoral and extraoral approaches have been described in the literature. The extraoral approach offers the benefit of enhanced exposure of the styloid process, but its disadvantage included increased surgical time, chance of injury to facial nerve, longer recovery period, and a disfiguring scar. The intraoral route is preferred by otolaryngologists because of its simplicity, reduced operative time, and better cosmetic outcome. However, the low accessibility, poor visualization of the surgical field, potential hemorrhage, and risk of deep neck space infections and iatrogenic injury to major neurovascular structures in the surrounding serve are some of the disadvantages associated with this approach. 5 In the present study, intraoral tonsillo-styloidectomy was the procedure of choice, and we modified the technique with the use of a microscope with a 400-mm lens. Microscope-assisted tonsillo-styloidectomy provided better visualization of the styloid process, its related anatomy, and of the location of bleeders for better hemostatic control. The lengths of the styloid processes resected varied from 0.5 cm to 3.8 cm ( Fig. 6 ). Hajare et al. 13 reported similar results, with resected lengths ranging from 0.6 cm to 3 cm. We had difficulty locating the styloid process in 5 cases due to anterior angulation, but no significant complications (bleeding, subcutaneous emphysema, deep neck space infections etc.) we observed intra- or postoperatively, due to the microscopic dissection technique and the good postoperative care.

All patients were routinely followed up for 12 weeks, when we observed a decrease in pain severity of more than two-thirds compared to the preoperative scores, so the treatment was considered successful. Four weeks postoperatively, 6 (50%) out of 12 patients were symptom-free (NRS-11 score of 0), and the other 6 (50%) patients presented mild pain (NRS-11 score of 1). By 12 weeks, 7 patients were symptom-free, while 5 still reported mild pain, and were prescribed NSAIDs and pregabalin (75mg/day) for 2 weeks, along with general instructions regarding jaw movement and hot fomentation for localized pain control. According to Härmä, 18 up to 20% of the patients may not experience complete symptom relief despite the removal of significant lengths of the styloid process.

Nevertheless, a larger sample size and long-term follow-up would be more helpful for a better assessment of the accuracy and validity of the surgical approach in the management of ES.

## Conclusion

The wide spectrum of symptoms and conditions in association with an elongated styloid process can often be misleading for the clinician. Hence, an otorhinolaryngologist should always keep ES in mind in cases of referred otalgia and throat pain. Pain relief in ES patients is the main goal of the treatment. With intraoral microscope-assisted tonsillo-styloidectomy, shortening and removal of the elongated styloid process is easier, and helps to avoid any intraoperative injury due to the better visualization. An underdiagnosed condition, ES requires more research to overcome the paucity of data and increase the knowledge of the treating doctors and surgeons on the latest techniques to better care for the patients and help improve their quality of life.
